# Endothelial cells secreted endothelin-1 augments diabetic nephropathy via inducing extracellular matrix accumulation of mesangial cells in ETBR^-/-^ mice

**DOI:** 10.18632/aging.101875

**Published:** 2019-03-29

**Authors:** Hong-hong Zou, Li Wang, Xiao-xu Zheng, Gao-si Xu, Yunfeng Shen

**Affiliations:** 1 Department of Nephrology, the Second Affiliated Hospital of Nanchang University, Nanchang, China; 2 Division of Renal Diseases and Hypertension, Department of Medicine, The George Washington University, Washington, DC 20052, USA; 3 Department of Endocrinology, the Second Affiliated Hospital of Nanchang University, Nanchang, China; * Equal contribution

**Keywords:** endothelin B receptor-deficient mice, endothelin-1, diabetic nephropathy, mesangial cells, endothelial cells, NF-kapapB

## Abstract

Endothelin B receptor (ETBR) deficiency may contribute to the progression of diabetic nephropathy (DN) in a streptozotocin (STZ) model, but the underlying mechanism is not fully revealed. In this study, STZ-diabetic ETBR^-/-^ mice was characterized by increased serum creatinine and urinary albumin, enhanced glomerulosclerosis, and upregulated ET-1 expression compared with STZ-diabetic WT mice. *In vitro*, HG conditioned media (CM) of ETBR^-/-^ GENs promoted mesangial cell proliferation and upregulated ECM-related proteins, and ET-1 knockout in GENs or inhibition of ET-1/ETAR in mesangial cell suppressed mesangial cell proliferation and collagen IV formation. In addition, ET-1 was over-expressed in ETBR^-/-^ GENs and was regulated by NF-kapapB pathway. ET-1/ETBR suppressed NF-kappaB to modulate ET-1 in GENs. Furthermore, ET-1/ETAR promoted RhoA/ROCK pathway in mesangial cells, and accelerated mesangial cell proliferation and ECM accumulation. Finally, *in vivo* experiments proved inhibition of NF-kappaB pathway ameliorated DN in ETBR^-/-^ mice. These results suggest that in HG-exposed ETBR^-/-^ GENs, suppression of ET-1 binding to ETBR activated NF-kappaB pathway, thus to secrete large amount of ET-1. Due to the communication between GENs and mesangial cells in diabetes, ET-1 binding to ETAR in mesangial cell promoted RhoA/ROCK pathway, thus to accelerate mesangial cell proliferation and ECM accumulation.

## Introduction

Diabetic nephropathy (DN), characterized by renal inflammation, urinary albumin, decreased glomerular filtration rate, glomerulosclerosis and tubulointerstitial fibrosis, is a leading cause of end-stage renal disease (ESRD) [1,2]. Thus, exploring the mechanisms that mediate DN is critical for the prevention and treatment strategies for alleviating DN. Mesangial cells (MC) are critical in maintaining mesangial matrix homeostasis, regulating glomerular filtration rate, and keeping normal glomerular function via producing cytokines, metalloproteinases and extracellular matrix (ECM) [3]. Researchers have found that mesangial cell proliferation and ECM accumulation were main contributing factors to glomerulosclerosis and tubulointerstitial fibrosis, which were important characters of DN [4,5]. In high-glucose (HG) condition, fibronectin, collagen IV, and plasminogen activator inhibitor were observed to be upregulated in mesangial cells [6]. Connective tissue growth factor (CTGF), a growth factor produced by activated mesangial cells, played a key role in the pathogenesis of DN and was also upregulated in DN [7]. Hence, glomerulosclerosis is closely associated with dysfunction of mesangial cells under HG condition.

Endothelin-1 (ET-1) is coded by EDN1 gene and mainly expressed by glomerulus endothelial cells (GENs) and plays an important role in DN [8,9]. ET-1 exerts its effects by binding to one of two endothelin receptor subtypes, namely Endothelin A Receptor (ETAR) and Endothelin B Receptor (ETBR). Studies have shown that ET-1 mediated the activation of ETAR in mesangial cells, and could elevate the expression of ECM-related genes [10]. Blocking ET-1/ETAR pathway protected patients from diabetes and chronic kidney disease through reducing albuminuria [11]. And ETAR blockade alone could also reduce albuminuria and restore endothelial glycocalyx coverage in diabetic nephropathy mice [12]. However, the effect of ET-1/ETBR pathway on DN is not clear. Olivia et al reported that ET-1 promoted glomerulosclerosis and podocyte loss by activation of ETAR and ETBR in podocyte, suggesting ETBR subtype was important in DN [13]. Mohamed et al found blocking ET-1/ETAR/ETBR pathway could also relieve DN, but the effect is less effective than ETAR-selective blockade [14]. Therefore, we speculated that ETBR blockade might play a promotion role in DN.

Researchers have found ETBR was mainly distributed in vascular and glomerular endothelial cells, whereas ETAR was mainly distributed in smooth muscle cells and mesangial cells [15]. Reports have shown that mesangial cells and endothelial cells interacted in the kidney [16,17], however, studies mainly focused on the ability of mesangial cells to regulate the synthesis of ET-1 by endothelial cells, the effect of endothelial cells on mesangial cells is rarely discussed. Based on ET-1 mainly expressed by endothelial cells and the crosstalk between endothelial cells and mesangial cells, we speculated that ETBR knockout in endothelial cells might have a vital function in mesangial cell proliferation and ECM accumulation.

Given the previous report of diabetic ETBR-deficient (ETBR^-/-^) rats caused progressive renal failure [18], we established streptozotocin (STZ)-diabetic ETBR^-/-^ mice model to explore the exact mechanism of ETBR^-/-^ mice in the acceleration of DN. Our results showed that STZ-diabetic ETBR^-/-^ mice enhanced glomerulosclerosis and had higher levels of renal damage signs (serum creatinine and urinary albumin) and increased mRNA and protein levels of ET-1 *in vivo*. Under high glucose condition, ETBR^-/-^ GENs secreted large amount of ET-1, thus to promote ECM accumulation of mesangial cells *in vitro*.

## RESULTS

### ETBR expression level was up-regulated in kidney tissue of DN

**Figure 1:**
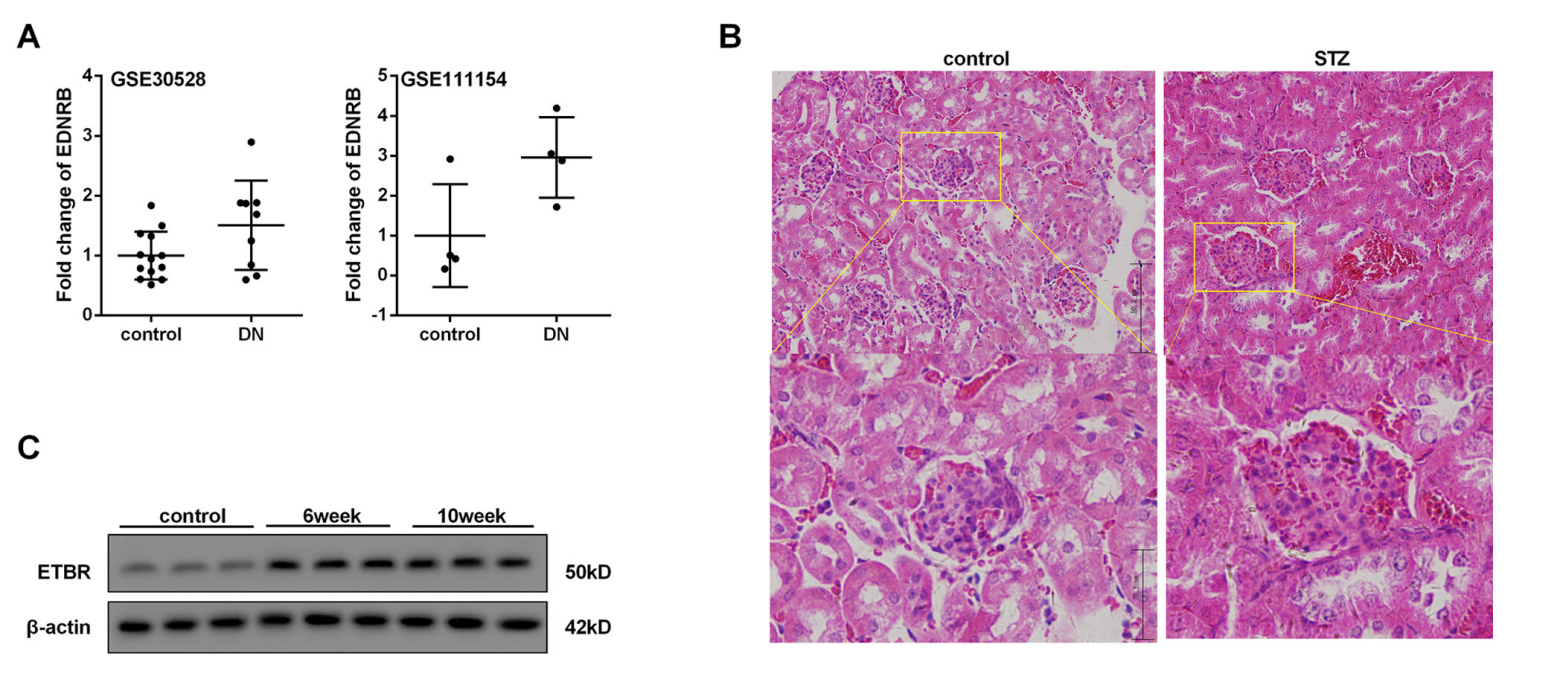
**ETBR expression level was up-regulated in kidney tissue of DN.** Ten weeks after the establishment of diabetic mice, the mice were sacrificed and kidneys were collected for further use. (**A**) mRNA levels of ETBR in kidney tissue from patients with DN (GEO database, GSE30528 and GSE111154) and healthy control. EDNRB, gene of ETBR. (**B**). HE staining of kidney tissue from STZ-diabetic mice and control mice was conducted to confirm the occurrence of diabetic nephropathy. (**C**). Protein level of ETBR in kidney tissue from STZ-diabetic mice at six and tenth weeks after the initial intraperitoneal injection of STZ. N=3.

According to the analysis of GEO database (GSE30528 and GSE111154), we found mRNA level of ETBR in kidney tissue from patients with DN was higher than that of healthy control (Figure 1A). HE staining showed increased glomerular volume, edema, necrosis and abscission of renal tubular epithelial cells in STZ-diabetic mice compared with control mice, which showed DN mice were successfully established (Figure 1B). Moreover, protein level of ETBR in kidney tissue from STZ-diabetic mice at the sixth and eighth weeks after the initial intraperitoneal injection of STZ was up-regulated than that of control mice (Figure 1C). Therefore, we focused on the effect of ETBR on DN, and used ETBR^-/-^ mice in the following experiments.

### Severer DN in ETBR^-/-^ mice

**Figure 2:**
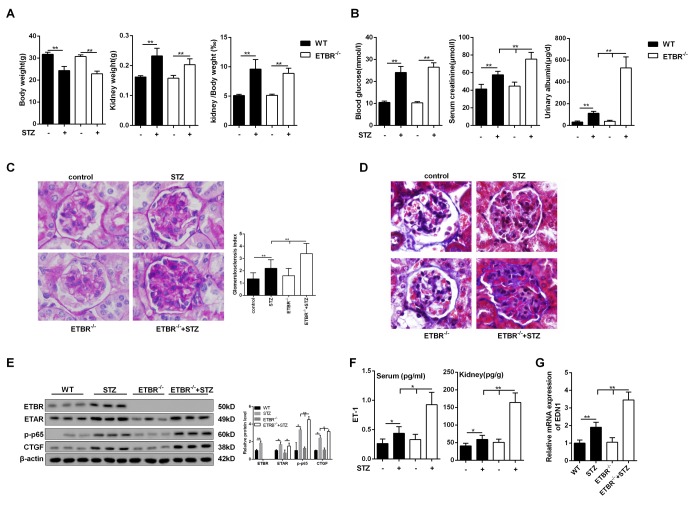
**Severer diabetic nephropathy in ETBR^-/-^ mice.** (**A**) Body weight and kidney weight were detected in control mice (WT), STZ-diabetic mice, ETBR^-/-^ mice and STZ-diabetic ETBR^-/-^ mice. **p<0.01, compared with control. N=5. (**B**) Serum glucose level, serum creatinine level and urinary albumin level were measure in control mice (WT), STZ-diabetic mice, ETBR^-/-^ mice and STZ-diabetic ETBR^-/-^ mice. **p<0.01, compared with control or STZ-diabetic WT mice. (**C**) Periodic acid-Schiff (PAS) staining of kidney tissues from control mice, STZ-diabetic WT mice, ETBR^-/-^ mice, and STZ-diabetic ETBR^-/-^ mice. **p<0.01, compared with control, STZ-diabetic WT, or ETBR^-/-^ mice. 1000×magnification. (**D**) MASSON staining of of kidney tissues from control mice, STZ-diabetic WT mice, ETBR^-/-^ mice, and STZ-diabetic ETBR^-/-^ mice. 1000×magnification. (**E**) Protein levels of ECM-related protein CTGF and p-p65 in control mice, STZ-diabetic WT mice, ETBR^-/-^ mice, and STZ-diabetic ETBR^-/-^ mice. *p<0.05, compared with WT, ETBR^-/-^, or STZ mice. **p<0.01, compared with WT, ETBR^-/-^, or STZ mice. N=3. (**F**-**G**) Serum ET-1/kidney ET-1 expressions and transcriptional level of EDN1 from kidney were detected in control mice, STZ-diabetic WT mice, ETBR^-/-^ mice, and STZ-diabetic ETBR^-/-^ mice. *p<0.05, **p<0.01, compared with WT, ETBR^-/-^, or STZ mice. Bars depict the mean ± SD. N=5.

STZ-diabetic mice had reduced body weight, increased kidney weight and increased kidney/body weight ratio (Figure 2A). And there were no significant differences in body weight and kidney weight between STZ-diabetic ETBR^-/-^ mice and STZ-diabetic WT mice (Figure 2A). As shown in Figure 2B, STZ-diabetic mice had higher serum glucose level, serum creatinine level and urinary albumin level than control mice. And serum creatinine and urinary albumin level in STZ-diabetic ETBR^-/-^ mice were significantly higher than that of STZ-diabetic WT mice (Figure 2B). PAS staining showed that enlargement of glomeruli was observed in STZ-diabetic mice, and enhanced glomerulosclerosis was present in STZ-diabetic ETBR^-/-^ mice, which was validated by glomerulosclerosis index (Figure 2C). MASSON staining showed collagen in the glomeruli was observably elevated in STZ-diabetic mice and STZ-diabetic ETBR^-/-^ mice (Figure 2D). Protein levels of ECM-related protein CTGF and p-p65 in STZ-diabetic ETBR^-/-^ mice was higher than STZ-diabetic WT mice (Figure 2E). ET-1 expression in STZ-diabetic ETBR^-/-^ mice was higher than STZ-diabetic WT mice at protein level (Figure 2F) and transcriptional level (Figure 2G).

### HG conditioned media (CM) of ETBR^-/-^ GENs promoted mesangial cell proliferation and ECM formation

**Figure 3:**
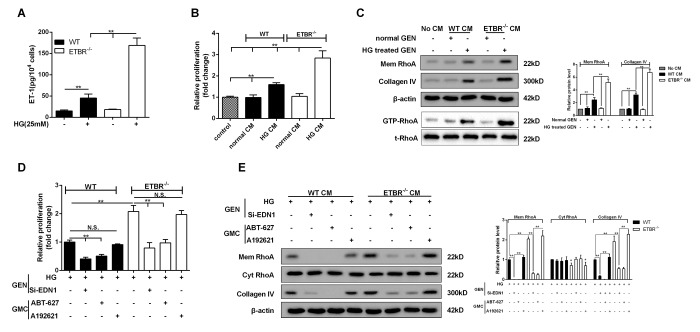
**HG conditioned media (CM) of ETBR^-/-^ GENs promoted mesangial cell proliferation and ECM formation.** (**A**) After 24 h of HG (25mM) treatment, ET-1 level in primary GENs of ETBR^-/-^ mice and WT mice was detected by ELISA. CM was collected for the culture of SV40 MES13 cells. **p<0.01 compared with control or HG WT. N=3. (**B**-**C**) WT or ETBR^-/-^ CM GEN was used to cultivate SV40 MES13 cells for 24 h. SV40 MES13 cells in control group was cultured in HG serum-free medium. The proliferation of SV40 MES13 cells was detected in control, WT normal CM, WT HG CM, ETBR^-/-^ normal CM and ETBR^-/-^ HG CM groups by MTT assay. RhoA level on SV40 MES13 cells membrane and Collagen IV secretionwere detected in control, WT normal CM, WT HG CM, ETBR^-/-^ normal CM and ETBR^-/-^ HG CM groups by western blot. GTP-RhoA level (the activity of Rho) was detected using Rhotekin RBD-agrose by Rho-pull down assay. **p<0.01 compared with control or normal WT CM or HG WT CM or normal ETBR^-/-^ CM. N=3. (**D**-**E**) GENs were transfected with 50 nM si-EDN1 for 18 h, and HG medium was used to culture GENs for 24 h, then the CM was collected for the culture of mesangial cells. 25 μM ABT-627 (blocking agent of ET-1/ETAR pathway) or 25μM A192621 (blocking agent of ET-1/ETBR pathway) was added to the medium for the culture of mesangial cells. The proliferation of mesangial cells was detected by MTT assay, and mem RhoA, cyt RhoA, collagen IV protein levels were detected by western blot. **p<0.01 compared with si-ET-1 or si-ET-1+ABT-627 or si-ET-1+A192621. Bars depict the mean ± SD. N=3.

After 24 h of HG treatment, protein level of ET-1 in primary GENs of ETBR^-/-^ mice was significantly higher than that of WT mice (Figure 3A). In this study, CM of ETBR^-/-^ GENs under normal or HG condition was used to cultivate mesangial cells. We found HG-treated CM of ETBR^-/-^ GENs promoted the proliferation of mesangial cells (Figure 3B). Western blotting assay showed HG-treated CM increased RhoA level on mesangial cell membrane and facilitated Collagen IV secretion, and the strengthen effect of ETBR^-/-^ CM on mesangial cell was stronger than WT CM (Figure 3C). Besides, GTP-RhoA level was increased in mesangial cells cultivated by HG-treated CM, suggesting HG-treated CM promoted RhoA activity (Figure 3C). Immunofluorescence assay showed RhoA diffused to cell membrane of mesangial cells after the treatment of ET-1 (Supplementary Figure 1). Later, we explored why HG-treated CM promoted mesangial cell proliferation and ECM formation. After ET-1 knockout in GENs, HG-treated CM significantly inhibited mesangial cell proliferation and Collagen IV formation, and the result was similar in ABT-627 group (blocking agent of ET-1/ETAR pathway) (Figure 3D and 3E). There was no significant difference in the result between A192621 group (blocking agent of ET-1/ETBR pathway) and ET-1 normal group. In addition, quantitative analysis showed that cyt RhoA expression was decreased when the expression of mem RhoA was significantly increased (Figure 3E). These results pointed out whether glomerulosclerosis in diabetic mice was related with ET-1 secretion, whether increased glomerulosclerosis index in ETBR^-/-^ mice was associated with higher ET-1 secretion in ETBR^-/-^ GENs, and why there was higher ET-1 secretion in ETBR^-/-^ GENs. These questions would be explored in the following experiments.

### ET-1 modulated mesangial cell proliferation and ECM through RhoA/Rho-kinase (ROCK) pathway

**Figure 4:**
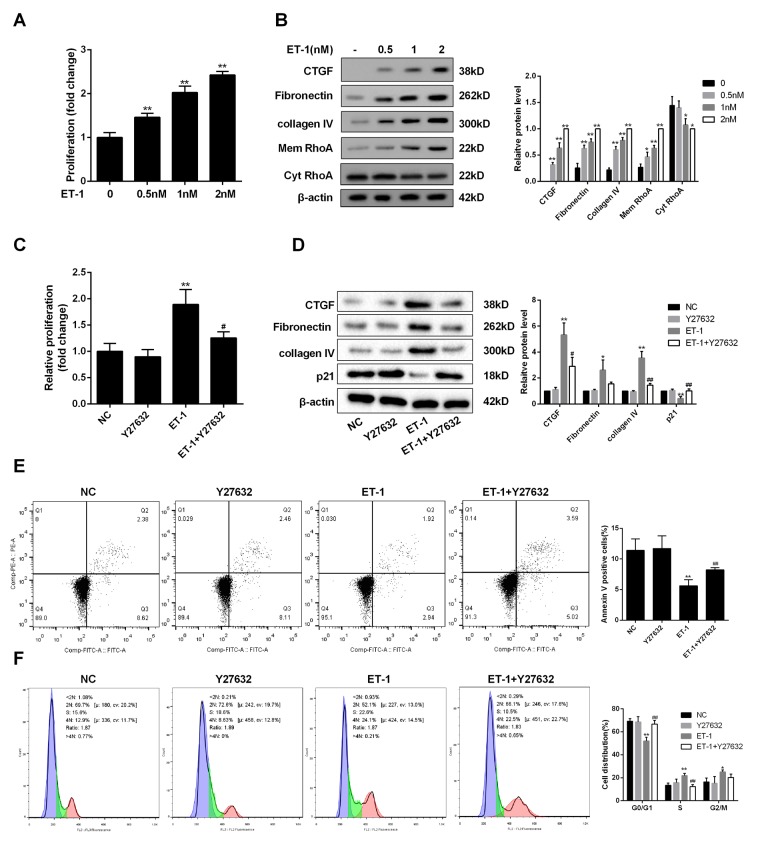
**ET-1 modulated mesangial cell proliferation and ECM through RhoA/ROCK pathway.** (**A**) Under HG serum-free condition, mesangial cell proliferation was detected after the treatment of ET-1 (0.5 nM, 1 nM, 2 nM) for 24 h. **p<0.01, compared with 0nM. (**B**) Under HG serum-free condition, ECM-related proteins (Collagen IV, Fibronectin and CTGF) and RhoA on mesangial cell membrane were detected after the treatment of ET-1 (0.5 nM, 1 nM, 2 nM) for 24 h. ET-1 (1nM, 2500pg/ml) was used for the following experiments. **p<0.01, compared with 0nM. (**C-F**) Under HG serum-free condition, mesangial cells were treated with ET-1 (1 nM), or ET-1 (1 nM)+Y-27632 (30 μM, RhoA/ROCK inhibitor) for 24 h. The proliferation, cell apoptosis, cell cycle and ECM-related proteins were detected in ET-1 and ET-1+Y-27632 groups using MTT, flow cytometry and western blot assay. **p<0.01, compared with ET-1. Bars depict the mean ± SD. N=3.

RhoA is mainly localized in the cytosol in un-stimulated cells. Due to the exchange of GDP/GTP, RhoA activation is usually associated with RhoA translation from the cytosol to the plasma membrane [20]. We therefore detected RhoA expression in the membrane. As shown in Figure 4A, mesangial cell proliferation was promoted with the increase of ET-1 concentration under HG serum-free condition. Under HG serum-free condition, ECM-related proteins (Collagen IV, Fibronectin and CTGF) and RhoA on mesangial cell membrane were also up-regulated with the increase of ET-1 concentration (Figure 4B). In addition, ET-1 significantly promoted the proliferation of mesangial cells, upregulated ECM-related proteins, and decreased the apoptosis of mesangial cells, and cell cycle was arrested at S and G2/M phases (Figure 4C, 4D, 4E and 4F). While Y-27632 (RhoA/ROCK inhibitor) reversed the effects of ET-1 on cell proliferation and cell apoptosis, moreover, cell cycle was arrested at G0/G1 phase (Figure 4C, 4D, 4E and 4F). However, there was no significant difference in ETAR expression between ET-1+Y27632 group and ET-1 group (data not shown).

### ET-1 promoted RhoA/ROCK pathway in mesangial cell through ETAR

**Figure 5:**
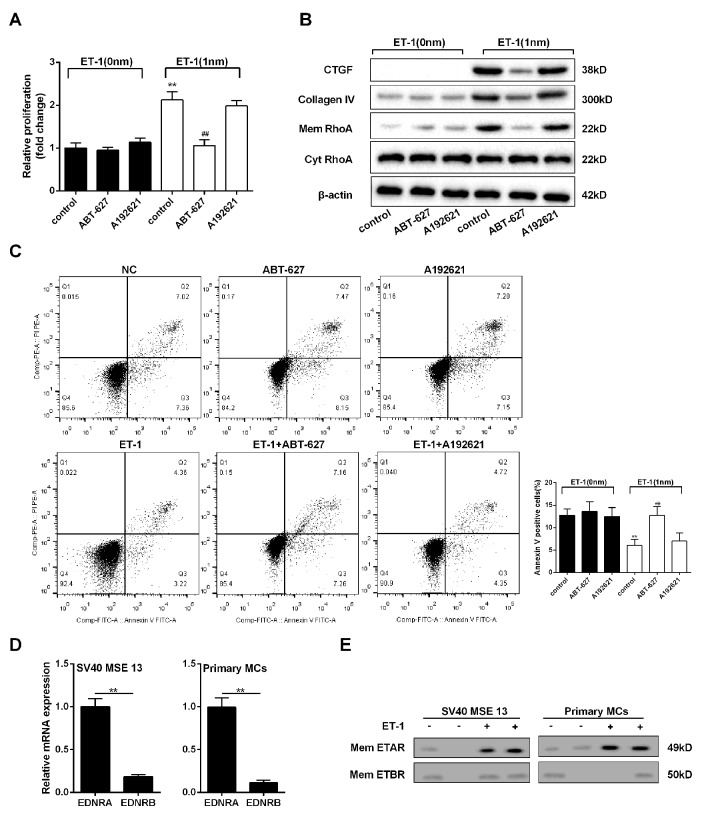
**ET-1 promoted RhoA/ROCK pathway in mesangial cells through ETAR.** Mesangial cells were treated with 1 nM ET-1, ABT-627(25μM, inhibitor of ETAR pathway) or A192621 (25μM, inhibitor of ETBR pathway) for 24 h. (**A**-**C**) Mesangial cell proliferation, RhoA/ROCK and ECM-related proteins, and cell apoptosis were detected in control, ABT-627, A192621, ET-1, ET-1+ ABT-627, ET-1+A192621 groups. **p<0.01 compared with ET-1 group. (**D**) Expression quantity from gene transcription level of EDNRA and EDNRB in mouse mesangial cells SV40 MSE 13 and mouse primary mesangial cells. **p<0.01 compared with ETAR. (**E**) ETAR and ETBR expressions on mesangial cell membrane were measured in ET-1 treated SV40 MSE 13 cells and primary mesangial cells. Bars depict the mean ± SD. N=3.

To explore whether ET-1 promoted RhoA/ROCK pathway through ETAR or ETBR, we administered ET-1/ETAR pathway inhibitor (ABT-627) and ET-1/ETBR pathway inhibitor (A192621) to mesangial cells. Under the treatment of ET-1, ABT-627 inhibited mesangial cell proliferation, RhoA/ROCK pathway and ECM-related proteins (CTGF and Collagen IV), and increased cell apoptosis, while inhibition of ETBR pathway didn’t affect cell growth, RhoA/ROCK pathway, ECM-related proteins and cell apoptosis (Figure 5A, 5B and 5C). Besides, expression quantity from gene transcription level of EDNRA in mesangial cell was higher than EDNRB (Figure 5D). In ET-1 treated mesangial cell, ETAR expression on mesangial cell membrane was increased, while ETBR expression didn’t change, which increased the probability of combination of ET-1 and ETAR. After ET-1/ETBR blockade by A192621 in mesangial cell, ET-1 treatment didn’t affect cell growth and ECM. While ET-1/ETAR blockade by ABT-627 inhibited cell growth and ECM, which indicated that ET-1/ETAR played the primary role in mesangial cell. Therefore, we speculated the effect of ET-1 stimulation on ETBR^-/-^ mesangial cell was similar as that on WT mesangial cell because ET-1/ETAR played the primary role in mesangial cell.

### ET-1 was over-expressed in ETBR^-/-^ GENs and was regulated by NF-kapapB pathway

**Figure 6:**
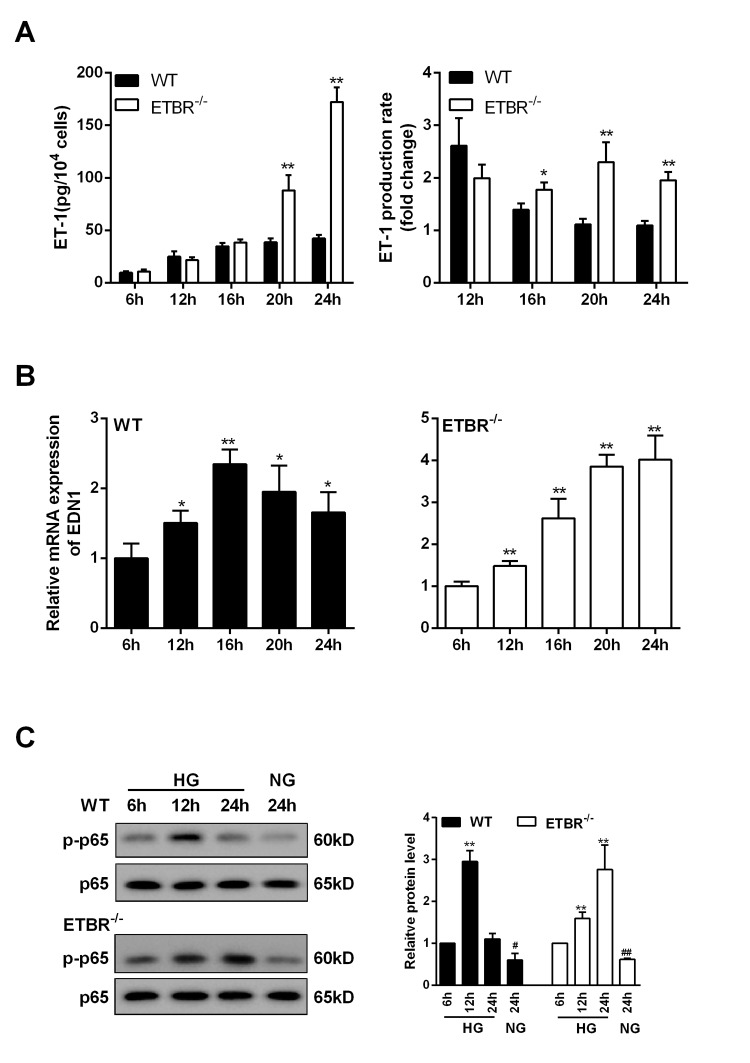
**ET-1 was overexpressed in ETBR knockout GENs and was regulated by NF-kapapB pathway.** GENs was cultured in HG medium, and the supernatant and GENs were collected at 6 h, 12 h, 16 h, 20 h, 24 h after cultivation. (**A**) Under the HG condition, ET-1 expression (in the supernatant)in WT and ETBR knockout GENs groups was detected at 6 h, 12 h, 16 h, 20 h, 24 hGENGEN . **p<0.01, compared with WT. There was significant difference in ET-1 production rate between ETBR knockout and WT GENs since 16 h, and the difference increased with time. ET-1 production rate (n)= ET-1(n)/ET-1(n-4). N represented the time point of sample collection. ET-1 represented the production of ET-1 in mesangial cells. *p<0.05 compared with WT. **p<0.01, compared with WT. (**B**) mRNA expressions of EDN1 in WT GENs and ETBR^-/-^ GENs groups were detected at 6 h, 12 h, 16 h, 20 h, 24 h. **p<0.01, compared with 6h. mRNA expression of ET-1 was increased in ETBR^-/-^ GENs within 24 h. **p<0.01, compared with 6h. (**C**) Protein levels of p-p65 in WT GENs and ETBR^-/-^ GENs groups were measured at 6 h, 12 h, 16 h, 20 h, 24 h. Bars depict the mean ± SD. N=3.

As shown in Figure 6A, ET-1 expression was increased with time in WT and ETBR^-/-^ GENs under the HG condition, and there was significant difference in ET-1 expression between ETBR^-/-^ and WT GENs until 20 h, which indicated that GENs grew fast between 16 h and 24 h. Besides, there was significant difference in ET-1 production rate between ETBR knockout and WT GENs since 16 h, and the difference increased with time (Figure 6A). We found mRNA expression of ET-1 in WT GENs reached the highest at 16 h (Figure 6B), and mRNA expression of ET-1 was increased in ETBR^-/-^ GENs within 24 h (Figure 6B). After silencing EDN1 in GENs, ETBR expression in the membrane of GENs was significantly decreased (data not shown), so, there might be a ligand independent effect of ETBR. Protein level of p-p65 in WT GENs reached the highest at 12 h, and protein level of p-p65 was increased in ETBR^-/-^ GENs within 24 h (Figure 6C). Therefore, we speculated whether NF-kappaB pathway was related with EDN1 mRNA level.

### p65 promoted EDN1 transcription, and ET-1/ETBR modulated ET-1 through NF-kappaB

**Figure 7:**
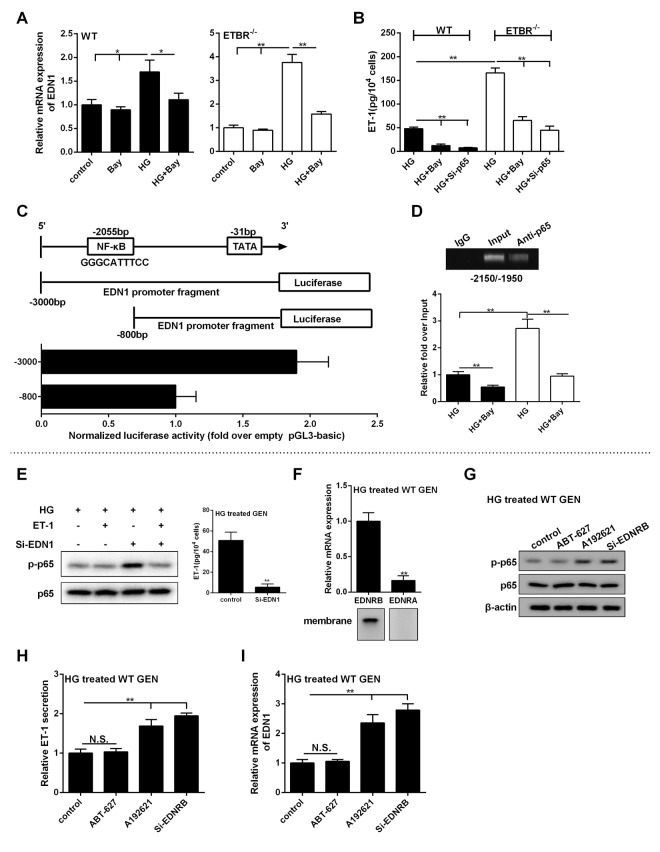
**p65 promoted the transcription of EDN1, and ET-1/ETBR modulated ET-1 through NF-kappaB.** (**A**) WT GENs or ETBR^-/-^ GENs were treated with HG or 10 μM Bay or HG+10 μM Bay for 24 h. mRNA expressions of EDN1 in WT or ETBR^-/-^ GENs were detected in control, Bay, HG, HG+Bay groups. *p<0.05, compared with control, Bay, or HG group. **p<0.01, compared with control, Bay, or HG group. (**B**) Extracellular secretion of ET-1 was detected in HG, HG+Bay and HG+si-p65 groups. **p<0.01, compared with HG or HG+Bay group. (**C**) CHO cells were co-transfected with p65-expressing vector and pGL3 vector carrying different promoters of EDN1, and empty pGL3 basic was used as control. After 48 h of transfection, EDN1 promoter activity was detected by dual-Luciferase Reporter Assay System. (**D**) WT or ETBR^-/-^ GENs were treated with HG or HG+10 μM Bay for 24 h. ChIP assay showed p65 could bind with the segments at this region in HG-treated GENs. Under HG condition, inhibition of NF-kappaB significantly decreased the binding efficiency of this region. **p<0.01, compared with HG group. (**E**) After GENs treated with HG for 6 h, 1 nM ET-1 was added into GENs and cultured for 18 h. So, GENs was treated with HG for 24 h in total. ET-1 secretion in GENs was detected in control and si-EDN1 group. p-p65 expression was detected in HG group, HG+ET-1 group, HG+si-EDN1 group, and HG+ET-1+si-EDN1 group. **p<0.01, compared with control group. (**F**) After WT GENs treated with HG for 24 h, mRNA level of ETAR and ETBR were detected in HG treated WT GENs. ETAR and ETBR protein levels were detected in the membrane of WT GENs. **p<0.01, compared with ETBR. (**G**-**I**) WT GENs were treated with HG, ABT-627 (25 μM), or A192621 (25 μM) for 24 h. Or WT GENs were transfected with 100 nM si-ETBR, then treated with HG for 24 h. p-p65 and p65 protein levels, ET-1 secretion level and EDN1 mRNA expression were detected in control, ABT-627, A192621 and si-ETBR groups.**p<0.01, compared with control or A192621 group. Bars depict the mean ± SD. N=3.

As shown in Figure 7A, Bay (NF-kappaB inhibitor) decreased mRNA expression of EDN1 in WT or ETBR^-/-^ GENs, which indicated EDN1 mRNA was regulated by NF-kappaB. We also found extracellular secretion of ET-1 was regulated by NF-kappaB (Figure 7B). The analysis of EDN1 showed it was at position 2055 bp upstream of the TSS, which contained RelA/p65 binding loci GGGCATTTCC [21]. Bioinformatics software JASPAR (http://jaspar.genereg.net) analyzed the promoter of EDN1 and found the binding loci of RelA gained the highest score. CHO cells were co-transfected with p65-expressing vector and pGL3 vector carrying different promoters of EDN1, and empty pGL3 basic was used as control. Luciferase assay showed NF-kappaB regulated the promoter of EDN1 at transcription level, and there existed p65 enhancer loci at the region of -3000 to -800 bp (Figure 7C). To confirm the binding loci of p65 at -2055 bp, ChIP assay showed p65 could bind with the segments at this region in HG-treated GENs (Figure 7D). Under HG condition, inhibition of NF-kappaB significantly decreased the binding efficiency of this region (Figure 7D). These findings indicated that NF-kappaB regulated the promoter of EDN1 at transcriptional level, thus to regulate ET-1 expression at mRNA and protein levels.

To determine the effect of ET-1 on p-p65 expression in GENs, GENs were cultivated in HG condition for 6 h, then 1 nm ET-1 (exogenous) was added into the medium. Eighteen hours later, protein level of p-p65 was detected (under HG condition for 24 h). As shown in Figure 7E, GENs normally secreted ET-1, so there was no significant change of p-p65 expression in HG group and HG+ET-1 group, which indicated that autocrine ET-1 was sufficient for regulating NF-kappaB pathway. So, the exogenous ET-1 had no significant change in p-p65 expression of NF-kappaB pathway. After EDN1 silencing, almost no ET-1 was secreted by GENs, and p-p65 expression in HG+si-EDN1 group was higher than that of HG group and HG+ET-1 group (Figure 7E). p-p65 expression was significantly downregulated in HG+ET-1+si-EDN1 group, indicating that ET-1 could decrease the activity of p65 under HG condition, which was opposite with previous reports [22,23]. We found that mRNA level of ETAR was significantly lower than ETBR, and ETAR protein in the membrane of GENs was not found under HG condition (Figure 7F). These findings suggested that ET-1 performed signal transduction through ETBR in GENs. Moreover, ABT-627 didn’t change p-p65 expression, whereas A192621 or si-ETBR significantly upregulated p-p65 expression (Figure 7G), which demonstrated our hypothesis that ET-1/ETBR mediated p-p65 expression. Our further study showed that ET-1/ETBR might regulate NF-kappaB pathway through eNOS (data not shown). NF-kappaB pathway regulated transcriptional activity of EDN1 mRNA (Figure 7A-D), therefore, blocking ET-1/ETBR suppressed p-p65 expression under HG condition to play a role in ET-1 clearance (Figure 7G-I).

According to the findings of Figure 6 and 7, it suggested that high glucose activated NF-kappaB pathway in GENs, which promoted the EDN1 transcription and ET-1 secretion, while the secreted ET-1 negatively fed back to p65 to inhibit EDN1 transcription through ETBR. Therefore, p65 was highly expressed 12h post HG treatment, yet decreased at the moment that ET-1 secretion reached approximately 40 pg/10^4^ cells, which appeared an intriguing balance between p65 and EDN1. And ETBR seemed to act as a clearance receptor to eliminate ET-1, and played an important role in ET-1-ETBR-p65-EDN1-ET-1-circulation in GENs. This balance circle was disrupted after knockdown of ETBR, and p65 and ET-1 secretion increased a lot in GENs without the inhibition of ETBR.

### Inhibition of NF-kappaB pathway ameliorated DN in ETBR^-/-^ mice in vivo

**Figure 8:**
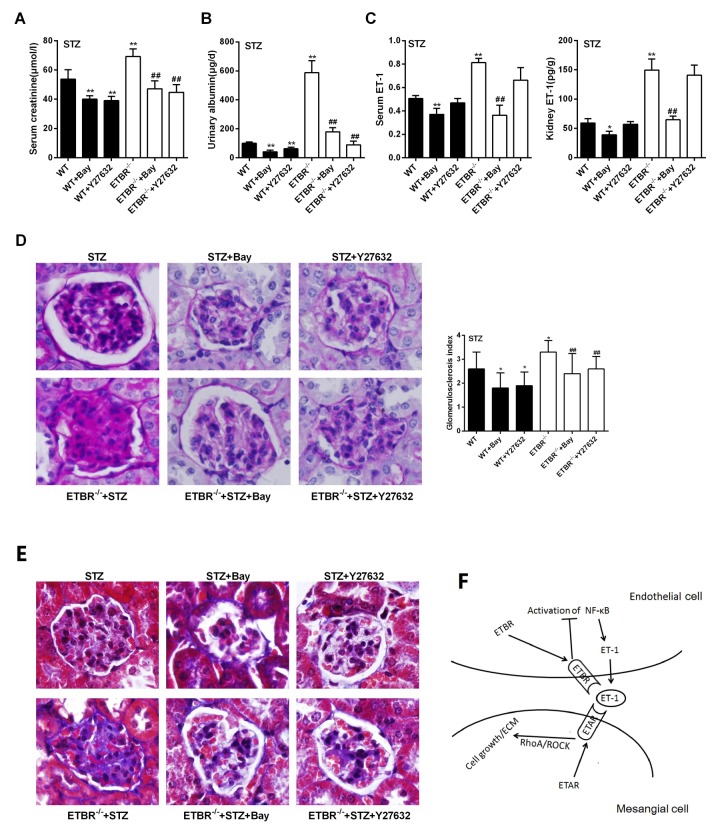
**Inhibition of NF-kappaB pathway ameliorated DN in ETBR-/- mice in vivo.** (**A**-**C**) C57BL/6 mice, ETBR^-/-^ mice were intraperitoneally injected with 50 mg/kg STZ every day for five days to establish STZ-diabetic mice model. Bay (1 mg/kg, Bay 11-7082) was dissolved in normal saline, and injected intraperitoneally twice a week between seventh and tenth weeks after STZ treatment. Y27632 (5 mg/kg, Rhoa/Rock inhibitor) was injected intraperitoneally twice a week between seventh and tenth weeks after STZ treatment. Serum creatinine, urinary albumin, serum ET-1 and kidney ET-1 were detected in WT, WT+Bay, WT+Y27632, ETBR^-/-^, ETBR^-/-^+Bay and ETBR^-/-^+Y27632 mice groups. **p<0.01 compared with WT or ETBR^-/-^. Bars depict the mean ± SD. N=6. (**D**) PAS staining showed that enlargement of glomeruli was observed in STZ-diabetic mice, and glomerulosclerosis was relieved in Bay and Y27632 treated WT or ETBR^-/-^ mice. **p<0.01 compared with STZ WT or STZ ETBR^-/-^. 1000×magnification. (**E**) MASSON staining showed that collagen was produced in glomeruli in STZ-diabetic mice, and the formation of collagen was relieved in Bay and Y27632 treated WT or ETBR^-/-^ mice. 1000×magnification. (**F**) Cascade diagram of signaling pathways.

Four indices (serum creatinine, urinary albumin, serum ET-1 and kidney ET-1) were detected in WT, WT+Bay, WT+Y27632, ETBR^-/-^, ETBR^-/-^+Bay and ETBR^-/-^+Y27632 mice groups. As shown in Figure 8A-C, serum creatinine and urinary albumin levels were significantly decreased in Bay and Y27632 treated WT or ETBR^-/-^ mice, and Bay significantly reduced ET-1 secretion, whereas there was no significant change in ET-1 secretion after Y27632 treatment. These findings indicated that NF-kappaB pathway regulated ET-1 expression. Although RhoA/Rock pathway had no significant regulatory effect on ET-1, it could inhibit the development of DN, and proved the results of *in vitro* experiments that RhoA/Rock was downstream molecule of ET-1, and GEN-secreted ET-1 regulated the proliferation and ECM of mesangial cell through RhoA/Rock pathway. Moreover, PAS staining showed that enlargement of glomeruli was observed in STZ-diabetic mice, and glomerulosclerosis was relieved in Bay and Y27632 treated WT or ETBR^-/-^ mice (Figure 8D). MASSON staining showed that collagen was produced in glomeruli in STZ-diabetic mice, and the formation of collagen was relieved in Bay and Y27632 treated WT or ETBR^-/-^ mice (Figure 8G). Cell signaling pathways were shown in Figure 8F.

## DISCUSSION

In the present study, we observed that STZ-diabetic ETBR^-/-^ mice had higher levels of renal damage signs (serum creatinine and urinary albumin), increased mRNA and protein levels of ET-1, enhanced glomerulosclerosis, and increased collagen in the glomeruli *in vivo*. Besides, protein levels of CTGF, ETAR and p-p65 were upregulated in STZ-diabetic ETBR^-/-^ mice. ET-1 expression in STZ-diabetic ETBR^-/-^ mice was higher than STZ-diabetic WT mice at protein level and transcriptional level. Under high glucose condition, ETBR^-/-^ GENs secreted large amount of ET-1, promoted mesangial cell proliferation, ECM accumulation of mesangial cell *in vitro*. We further demonstrated that ET-1 over-expression in ETBR^-/-^ GENs was regulated by NF-kapapB pathway, and found ET-1/ETBR suppressed NF-kappaB to modulate ET-1. We also proved ET-1/ETAR promoted RhoA/ROCK pathway in mesangial cell, thus to modulate mesangial cell proliferation and ECM accumulation. Based on the crosstalk between GENs and mesangial cell, our results first revealed that in STZ-diabetic ETBR^-/-^ mice, ET-1/ETBR was suppressedand NF-kappaB pathway was activated in GENs, thus to secrete large amount of ET-1. Then, ET-1 binding to ETAR in mesangial cell, so RhoA/ROCK pathway was promoted, thus to accelerate mesangial cell proliferation and ECM accumulation.

NF-kappaB, a transcription factor that has five subunits, namely p50, p52 RelA/p65, c-Rel and RelB, plays a critical role in inflammatory process and metabolic disease [24]. It has been reported that high blood glucose, urinary albumin, angiotensin II could contribute to NF-kappaB activation [25,26]. Evidences have shown that NF-kappaB activation in endothelial cells exerted a vital role in DN. Suppression of NF-kappaB attenuated HG-induced endothelial cell inflammation [27]. Advanced glycation end products increased NF-kappaB-binding activity to the promoter of ET-1 thus to increase ET-1 expression [28]. Researchers reported that C-peptide protected DN by preventing NF-kappaB from recruiting p300 and binding to the inos promoter [29]. Kolati et al demonstrated that NF-kappaB inhibitor BAY 11-7082 ameliorated DN by inhibiting renal inflammation and oxidative stress [30]. Our results showed NF-kappaB regulated promoter of ET-1 at transcription level, and the expression of p65, a constituent of NF-kappaB, was remarkably upregulated after ET/ETBR inhibition. This study first revealed suppression of ET-1/ETBR largely upregulated ET-1 expression through NF-kappaB pathway in ETBR^-/-^ endothelial cells exposed to HG, which will enrich the literature and provide theoretical basis for the treatment of DN.

RhoA is a protein that cycle between active and inactive forms depending on binding to GTP or GDP [31]. ROCK, a serine/threonine kinase, is a downstream target of ROCK. Studies have shown that HG activated RhoA/ROCK pathway in mesangial cell, which contributed to the progression of DN [32,33]. Researchers proved ET-1 binding to ETAR was involved in CTGF synthesis via RhoA/ROCK pathway in vascular smooth muscle cells [34]. Lee et al further discovered that over-expression of ET-1 binding to ETAR facilitated RhoA/ROCK pathway to induce collagen synthesis and proteinuria in hypertensive rats [35]. However, supporting evidences are still lacked for the acceleration effect of ET-1 binding to ETAR on mesangial cell proliferation and ECM accumulation via RhoA/ROCK pathway. Our result showed that membrane ETAR was highly expressed after ET-1 treatment, and inhibition of ET-1/ETAR suppressed mesangial cell proliferation, decreased CTGF and collagen IV protein levels, and inhibited RhoA/ROCK pathway. Moreover, RhoA/ROCK inhibitor suppressed mesangial cell proliferation, decreased ECM accumulation and promoted cell apoptosis with the treatment of ET-1. Therefore, we first proved that under HG condition, large amount of ET-1 binding to ETAR accelerated mesangial cell proliferation and ECM accumulation through promoting RhoA/ROCK pathway.

In conclusion, we have demonstrated that in HG exposed ETBR^-/-^ endothelial cells, suppression of ET-1 binding to ETBR activated NF-kappaB pathway, thus to secrete large amount of ET-1. Due to the crosstalk between GENs and mesangial cell, ET-1 binding to ETAR in MC promoted RhoA/ROCK pathway, thus to accelerate mesangial cell proliferation and ECM accumulation. Therefore, STZ-diabetic ETBR^-/-^ mice accelerated the progression of DN.

## MATERIALS AND METHODS

### Animals and establishment of diabetic mice

Male C57BL/6 (wild-type, WT) mice and ETBR^-/-^ mice (aged 7 weeks) were intraperitoneally injected with 50 mg/kg STZ (Sigma, USA) every day for five days to establish STZ-diabetic mice model. Blood was collected by cutting tails, and diabetic mice were confirmed two weeks after the initial intraperitoneal injection with the criteria of a blood glucose >16 mmol/L. Urine was collected in metabolic cages (Hazleton Systems Inc., Aberdeen, USA) in 24h. WT mice and ETBR^-/-^ mice were used as control and received an intraperitoneal injection of 0.1 M citrate buffer (pH 4.5) every day. All mice were kept in metabolic cages and had a standard diet (0.2% sodium) with no limitation to water. Before killing, blood was collected from retro-orbital vein plexus, after 4 °C for the night and centrifugation for 10 min at 3000×g, serum was obtained and stored at -20°C for the detection of creatinine and ET-1. Urine was also collected from the mice for the detection of urinary albumn. Ten weeks later, all the mice were sacrificed to detect the indices. Kidneys were collected for further use and weighed. The left kidneys were kept in nitrogen for qRT-PCR, WB, ELISA detection. The right kidneys were fixed in 4% formaldehyde solution for PAS and MASSON staining. The animal study was approved by the Ethic Committee of the Second Affiliated Hospital of Nanchang University.

*In vivo* experiment, C57BL/6 mice, ETBR^-/-^ mice (six groups, n=6/group) were intraperitoneally injected with 50 mg/kg STZ every day for five days to establish STZ-diabetic mice model. Diabetic mice were confirmed two weeks after the initial intraperitoneal injection with the criteria of a blood glucose >16 mmol/L. Bay 11-7082 (1mg/kg) was injected intraperitoneally twice a week between seventh and tenth weeks after STZ treatment. Y27632 (5 mg/kg, Rhoa/Rock inhibitor) was injected intraperitoneally twice a week between seventh and tenth weeks after STZ treatment. Normal saline was used as vehicle control.

### Serum and urine detection

The levels of serum glucose, serum creatinine, urinary albumin were detected by blood glucose meter (Roche, Switzerland), Inosine Assay Kit (Nanjing Jiancheng Biological Engineering Institute, China), Albumin (Mouse) Elisa Kit according to the manufacturer’s instructions (Abnova, Taiwan). ET-1 concentration was measured by ET-1 ELISA kit (Shanghai Jingkang Biotechnology, China) according to the manufacturer’s instructions.

### Renal morphology assessment

After fixation of the kidney, the slices were embedded in paraffin. Sections of 3μm were stained by periodic acid-Schiff (PAS) and hematoxylin eosin (HE) and MASSON’s trichrome to identify kidney structure. MASSON staining of kidney tissue was conducted by Trichrome stain (MASSON) Kit (Solarbio, China). Glomerulosclerosis was defined by the presence of PAS-positive material within the glomeruli. Twenty glomeruli specimens in each group were used to observe the glomerulosclerosis by Periodic acid-Schiff (PAS) Kit (Solarbio, China). The scoring guidelines of the proportion of PAS-positive material within each glomerulus are: 1, a proportion <25%; 2, a proportion of 25%-50%; 3, a proportion of 50%-75%; 4, a proportion > 75%. The average score assigned to all glomeruli was defined as glomerulosclerosis index.

### Cell culture and transfection

Primary glomerulus endothelial cells (GENs) were obtained from the following procedures: pronase (Roche, Switzerland) followed by collagenase (Roche, Switzerland) were used for in situ perfusion of the liver from ETBR^-/-^ mice. Discontinuous density gradient of Accudenz (Accurate Chemical and Scientific, Canada) was used to layer cell suspensions. GENs were present in the lower layer and further purified by centrifugal elutriation (18 ml/min flow). Cells were cultured in DMEM/F-12 (Gibco, USA) containing 20% fetal bovine serum (Gibco, USA). GENs after the third passage were probed with anti-CD31 (BD, USA) to confirm the purity of primary GENs was greater than 95%.

Mouse primary mesangial cells were obtained from cortical renal tissue using a sieving technique. Briefly, cortical renal tissues were pressed and rinsed with Hank’s salt solution through 60 steel mesh screen (pore size 250 μm), 150 mesh screen (pore size 150 μm), 200 mesh screen (pore size 75 μm). After 30 min of collagenase digestion, mesangial cells can be grown through plating of the dissociated cells into culture [19].

Mouse glomerular mesangial cell line SV40 MSE13 was purchased from Cell bank of Chinese Academy of Sciences (Shanghai, China) and cultured in DMEM/F-12 (Gibco, USA) containing 10% FBS (Gibco, USA), 14mM HEPES (Gibco, USA), 150mg/L L-glutamine (Sinopharm Chemical Reagent, China) and 1.5g/L NaHCO_3_ (Sinopharm Chemical Reagent, China) in 5% CO_2_ incubator under 37°C.

Cells were seeded into a culture plate and grown to 80% confluence for cell transfection. si-ET-1, si-p65, and si-ETBR were synthesized by GENECHEM (Shanghai, China), and were transfected into cells using Lipofectamine 2000 (Invitrogen, USA).

### Western blot

Renal cortex was homogenized and extracted in a cold buffer containing 0.1 mol/l Tris (hydroxymethyl) aminomethane HCl. The tissue extracts were then partially purified by ethanol extraction. Proteins from GENs or mesangial cells were isolated from using RIPA buffer (Thermo Scientific, USA). 50 μg of protein samples were isolated in 12% SDS-polyacrylamide gel electrophoresis (SDS-PAGE), and transferred to polyvinylidene difluoride (PVDF) membranes (Invitrogen, USA) by electroblotting. The membranes were blocked in 5% non-fat dried milk for 60 min at room temperature. For the detection of RhoA, the membrane and cytoplasm proteins were extracted from mesangial cells using Membrane and Cytosol Protein Extraction Kit (Beyotime Biotechnology), then membrane and cytosolic proteins were separated on SDS-PAGE. The membranes were probed with first primary antibody anti-ETBR (Abcam, USA), anti-ETAR (Abcam, USA), anti-p-p65 (Invitrogen, USA), anti-p65 (Invitrogen, USA), anti-CTGF (Abcam, USA), anti-RhoA (Abcam, USA), anti-collagen IV (Abcam, USA), anti-Fibronectin (Abcam, USA), anti-p21 (Abcam, USA) and anti-β-actin (Invitrogen, USA) and incubated at 4°C overnight. After washing with PBST, membrane was cultivated with secondary antibody for 60 min at room temperature. β-actin was used as internal control.

### Quantitative real-time PCR (qRT-PCR)

To quantify mRNA expression of EDN1, EDNRA and EDNRB, we conducted qRT-PCR. Total RNA from kidney tissue, GENs or mesangial cells were isolated by TRIzol Reagent (Invitrogen, USA) according to manufacturer’s instructions, and was transcribed to cDNA with iScript cDNA Synthesis kit (Bio-Rad, USA). PCR was performed as follows: 94°C for 5 min, then 35 cycles of denaturation at 94°C for 30 s, annealing at 64°C for 30 s and extension at 72°C for 120 s at QuantStudio 3 RCR Real-Time PCR systems (Applied Biosystems, USA). The relative EDN1, EDNRA and EDNRB expressions were determined by via the comparative 2-^ΔΔCq^ method. The primer sequences were provided as follows: EDN1, F: 5’-CTTCTGCCACCTGGACATCATCTG-3’, R: 5’-GGAACGCTTGGACCTGGAAGAAC-3’; ETAR (EDNRA), F: 5’-GTCCTGCCTCTGTTGCTGTTGTC-3’, R: 5’-TCCGTTCCGTGTTGTGGTTGTTC-3’; ETBR (EDNRB), F: 5’-CCAAGCCACGCTGTCACTTCTC-3’, R: 5’-GAGGAACGCATCAGACTGGAGTTG-3’.

### MTT assay

3-(4,5-dimethylthiazol-2-yl)-2,5-diphenyltetrazolium bromide (MTT) assay was used to detect the proliferation of mesangial cells. 1× 10^5^ mesangial cells were seeded into a 96-well plate. After overnight incubation with different treatment, 20 μl MTT (5 mg/mL; Invitrogen, USA) was added to each well and cultured for 2 h. Then, cells were lysed using dimethylsulfoxide (150 μl/well; Sinopharm Chemical Reagent, China). The optical density was read at 570 nm.

### RhoA pull-down assay

For detection of RhoA activity, RhoA pull-down assay was used to detect the level of GTP-RhoA. Proteins were incubated with Rhotekin Rho binding domain (RBD)-agrose (Upstate,USA) for 30 min at 4°C. GTP-RhoA was analyzed by Western blotting with specific rabbit anti-RhoA antibody (Santa Cruz, USA) and incubated with HRP-conjugated goat anti-rabbit IgG.

### Apoptosis assay

After washing with cold phosphate-buffered saline (PBS), 5 μl of annexin-V-FITC (Beyotime Biotechnology, China) was added to the mesangial cells and incubated at room temperature for 15 min. Then, 10 μl propidium iodide (PI) was added before flow cytometry analysis. Apoptosis were measured using a FACS Calibur flow cytometer (BD, USA) and analyzed by Cell Quest pro software.

### Cell cycle analysis

After 72 h of treatment, mesangial cells were harvested by trypsinization and washed with cold PBS for two times. Then, mesangial cells were fixed with 75% alcohol for 12 h at 4°C. After washing with cold PBS, cells were treated with 50 ug/mL RNase for 30 min at 37 °C, then stained with 50 μg/mL PI for 30 min at 4 °C in the dark before being analyzed using a FACS Calibur flow cytometer (BD, USA). Cells (2×10^6^) were detected for each sample. Cell cycle was analyzed by FlowJo software.

### Reporter gene assay

p65 cDNA was inserted into pcDNA3.1 vector (Invitrogen) using Xho I enzyme (F: 5’-CCCTCGAGATGGACGATCTGTTTCCCCT-3’) and Hind III enzyme (R: 5’-CCCAAGCTTTTAGGAGCTGATCTGACTC-3’) to construct pcDNA3.1-p65 plasmid. pcDNA3.1-p65 plasmid and pGL3-basic-luciferase vectors containing EDN1 promoter fragments differing in length (-3000 bp: forward primer with XhoI site, 5’-CCCTCGAGTAGCTATCTATTAATATGCAGCC-3’, reverse primer with HindIII site, 5’-CCCAAGCTTGACTCGGACAGTTCTCCG-3’; -800 bp: forward primer with XhoI site, 5’-CCCTCGAGCTGGGCACAAGAAATATTGG-3’, reverse primer with HindIII site, 5’-CCCAAGCTTGACTCGGACAGTTCTCCG-3’) were co-transfected into CHO cells (Chinese hamster ovary). Empty pGL3-basic was used as control. Firefly luciferase reporter was used to measure EDN1 promoter activity. After 48 h of transfection, dual-Luciferase Reporter Assay System (Promega, USA) was used to measure reporter activities.

### Chromatin immunoprecipitation (ChIP) assay

ChIP assay was performed using Pierce Magnetic ChIP Kit (ThermoFisher, CA, USA) according to the manufacturer’s instruction. The primer sequences used for EDN1 were F: 5’-TCCCTGATGCTGTGGCCATCGC-3’ and R: 5’-AGTACTCCCCTCCCCCCCACA-3’. Cross-linked chromatin was sonicated into -2150 to -1950 bp fragments. Then, the chromatin was immunoprecipitated using an anti-p65 antibody. qRT-PCR was conducted to detect the binding efficiency of this region according to the method described above.

### Statistical analysis

SPSS software (version 18.0) was used for data analysis, and the result was expressed as mean ± standard deviation (SD). One-way ANOVA and t test were used for the data analysis, with p < 0.05 considered statistically significant.

## Supplementary Material

Supplementary Figure

## References

[1] Hinden L, Udi S, Drori A, Gammal A, Nemirovski A, Hadar R, Baraghithy S, Permyakova A, Geron M, Cohen M, Tsytkin-Kirschenzweig S, Riahi Y, Leibowitz G, et al. Modulation of Renal GLUT2 by the Cannabinoid-1 Receptor: Implications for the Treatment of Diabetic Nephropathy. J Am Soc Nephrol. 2018; 29:434–48. 10.1681/ASN.2017040371 29030466 PMC5791066

[2] Badal SS, Danesh FR. Diabetic Nephropathy: Emerging Biomarkers for Risk Assessment. Diabetes. 2015; 64:3063–65. 10.2337/db15-0738 26294427 PMC4876692

[3] Abboud HE. Mesangial cell biology. Exp Cell Res. 2012; 318:979–85. 10.1016/j.yexcr.2012.02.025 22414873

[4] Han F, Xue M, Chang Y, Li X, Yang Y, Sun B, Chen L. Triptolide Suppresses Glomerular Mesangial Cell Proliferation in Diabetic Nephropathy Is Associated with Inhibition of PDK1/Akt/mTOR Pathway. Int J Biol Sci. 2017; 13:1266–75. 10.7150/ijbs.20485 29104493 PMC5666525

[5] Liu HF, Liu H, Lv LL, Ma KL, Wen Y, Chen L, Liu BC. CCN3 suppresses TGF-β1-induced extracellular matrix accumulation in human mesangial cells in vitro. Acta Pharmacol Sin. 2018; 39:222–29. 10.1038/aps.2017.87 28858296 PMC5800473

[6] Qin G, Zhou Y, Guo F, Ren L, Wu L, Zhang Y, Ma X, Wang Q. Overexpression of the FoxO1 Ameliorates Mesangial Cell Dysfunction in Male Diabetic Rats. Mol Endocrinol. 2015; 29:1080–91. 10.1210/me.2014-1372 26029993 PMC5414709

[7] Wahab N, Cox D, Witherden A, Mason RM. Connective tissue growth factor (CTGF) promotes activated mesangial cell survival via up-regulation of mitogen-activated protein kinase phosphatase-1 (MKP-1). Biochem J. 2007; 406:131–38. 10.1042/BJ20061817 17489738 PMC1948989

[8] Nelson J, Bagnato A, Battistini B, Nisen P. The endothelin axis: emerging role in cancer. Nat Rev Cancer. 2003; 3:110–16. 10.1038/nrc990 12563310

[9] Gagliardini E, Zoja C, Benigni A. Et and diabetic nephropathy: preclinical and clinical studies. Semin Nephrol. 2015; 35:188–96. 10.1016/j.semnephrol.2015.03.003 25966350

[10] Yang C, Ling H, Zhang M, Yang Z, Wang X, Zeng F, Wang C, Feng J. Oxidative stress mediates chemical hypoxia-induced injury and inflammation by activating NF-κb-COX-2 pathway in HaCaT cells. Mol Cells. 2011; 31:531–38. 10.1007/s10059-011-1025-3 21533553 PMC3887613

[11] Andress DL, Coll B, Pritchett Y, Brennan J, Molitch M, Kohan DE. Clinical efficacy of the selective endothelin A receptor antagonist, atrasentan, in patients with diabetes and chronic kidney disease (CKD). Life Sci. 2012; 91:739–42. 10.1016/j.lfs.2012.01.011 22326504

[12] Boels MG, Avramut MC, Koudijs A, Dane MJ, Lee DH, van der Vlag J, Koster AJ, van Zonneveld AJ, van Faassen E, Gröne HJ, van den Berg BM, Rabelink TJ. Atrasentan Reduces Albuminuria by Restoring the Glomerular Endothelial Glycocalyx Barrier in Diabetic Nephropathy. Diabetes. 2016; 65:2429–39. 10.2337/db15-1413 27207530

[13] Lenoir O, Milon M, Virsolvy A, Hénique C, Schmitt A, Massé JM, Kotelevtsev Y, Yanagisawa M, Webb DJ, Richard S, Tharaux PL. Direct action of endothelin-1 on podocytes promotes diabetic glomerulosclerosis. J Am Soc Nephrol. 2014; 25:1050–62. 10.1681/ASN.2013020195 24722437 PMC4005294

[14] Saleh MA, Pollock JS, Pollock DM. Distinct actions of endothelin A-selective versus combined endothelin A/B receptor antagonists in early diabetic kidney disease. J Pharmacol Exp Ther. 2011; 338:263–70. 10.1124/jpet.111.178988 21471190 PMC3126640

[15] Neuhofer W, Pittrow D. Endothelin receptor selectivity in chronic kidney disease: rationale and review of recent evidence. Eur J Clin Invest. 2009; 39:50–67, (Suppl 2). 10.1111/j.1365-2362.2009.02121.x 19335747

[16] Dimke H, Maezawa Y, Quaggin SE. Crosstalk in glomerular injury and repair. Curr Opin Nephrol Hypertens. 2015; 24:231–38. 10.1097/MNH.0000000000000117 25887901 PMC4465999

[17] López-Ongil S, Díez-Marqués ML, Griera M, Rodríguez-Puyol M, Rodríguez-Puyol D. Crosstalk between mesangial and endothelial cells: angiotensin II down-regulates endothelin-converting enzyme 1. Cell Physiol Biochem. 2005; 15:135–44. 10.1159/000083646 15665524

[18] Pfab T, Thöne-Reineke C, Theilig F, Lange I, Witt H, Maser-Gluth C, Bader M, Stasch JP, Ruiz P, Bachmann S, Yanagisawa M, Hocher B. Diabetic endothelin B receptor-deficient rats develop severe hypertension and progressive renal failure. J Am Soc Nephrol. 2006; 17:1082–89. 10.1681/ASN.2005080833 16495378

[19] Kreisberg JI, Karnovsky MJ. Glomerular cells in culture. Kidney Int. 1983; 23:439–47. 10.1038/ki.1983.40 6341686

[20] Takaishi K, Sasaki T, Kameyama T, Tsukita S, Tsukita S, Takai Y. Translocation of activated Rho from the cytoplasm to membrane ruffling area, cell-cell adhesion sites and cleavage furrows. Oncogene. 1995; 11:39–48. 7624130

[21] Cookson VJ, Waite SL, Heath PR, Hurd PJ, Gandhi SV, Chapman NR. Binding loci of RelA-containing nuclear factor-kappaB dimers in promoter regions of PHM1-31 myometrial smooth muscle cells. Mol Hum Reprod. 2015; 21:865–83. 10.1093/molehr/gav051 26405173

[22] Cianfrocca R, Tocci P, Semprucci E, Spinella F, Di Castro V, Bagnato A, Rosanò L. β-Arrestin 1 is required for endothelin-1-induced NF-κB activation in ovarian cancer cells. Life Sci. 2014; 118:179–84. 10.1016/j.lfs.2014.01.078 24530737

[23] von Brandenstein MG, Ngum Abety A, Depping R, Roth T, Koehler M, Dienes HP, Fries JW. A p38-p65 transcription complex induced by endothelin-1 mediates signal transduction in cancer cells. Biochim Biophys Acta. 2008; 1783:1613–22. 10.1016/j.bbamcr.2008.04.003 18457675

[24] Baker RG, Hayden MS, Ghosh S. NF-κB, inflammation, and metabolic disease. Cell Metab. 2011; 13:11–22. 10.1016/j.cmet.2010.12.008 21195345 PMC3040418

[25] Ding LH, Liu D, Xu M, Wu M, Liu H, Tang RN, Ma KL, Chen PS, Liu BC. TLR2-MyD88-NF-κB pathway is involved in tubulointerstitial inflammation caused by proteinuria. Int J Biochem Cell Biol. 2015; 69:114–20. 10.1016/j.biocel.2015.10.014 26485683

[26] Fei Y, Sun L, Yuan C, Jiang M, Lou Q, Xu Y. CFTR ameliorates high glucose-induced oxidative stress and inflammation by mediating the NF-κB and MAPK signaling pathways in endothelial cells. Int J Mol Med. 2018 10.3892/ijmm.2018.3547 29512777

[27] Yang J, Han Y, Chen C, Sun H, He D, Guo J, Jiang B, Zhou L, Zeng C. EGCG attenuates high glucose-induced endothelial cell inflammation by suppression of PKC and NF-κB signaling in human umbilical vein endothelial cells. Life Sci. 2013; 92:589–97. 10.1016/j.lfs.2013.01.025 23395866

[28] Adamopoulos C, Piperi C, Gargalionis AN, Dalagiorgou G, Spilioti E, Korkolopoulou P, Diamanti-Kandarakis E, Papavassiliou AG. Advanced glycation end products upregulate lysyl oxidase and endothelin-1 in human aortic endothelial cells via parallel activation of ERK1/2-NF-κB and JNK-AP-1 signaling pathways. Cell Mol Life Sci. 2016; 73:1685–98. 10.1007/s00018-015-2091-z 26646068 PMC11108501

[29] Li Y, Li X, He K, Li B, Liu K, Qi J, Wang H, Wang Y, Luo W. C-peptide prevents NF-κB from recruiting p300 and binding to the inos promoter in diabetic nephropathy. FASEB J. 2018; 32:2269–79. 10.1096/fj.201700891R 29229684

[30] Kolati SR, Kasala ER, Bodduluru LN, Mahareddy JR, Uppulapu SK, Gogoi R, Barua CC, Lahkar M. BAY 11-7082 ameliorates diabetic nephropathy by attenuating hyperglycemia-mediated oxidative stress and renal inflammation via NF-κB pathway. Environ Toxicol Pharmacol. 2015; 39:690–99. 10.1016/j.etap.2015.01.019 25704036

[31] Etienne-Manneville S, Hall A. Rho GTPases in cell biology. Nature. 2002; 420:629–35. 10.1038/nature01148 12478284

[32] Peng F, Wu D, Gao B, Ingram AJ, Zhang B, Chorneyko K, McKenzie R, Krepinsky JC, and F. Peng. D. Wu, B. Gao, A.J. Ingram, B. Zhang, K. Chorneyko, R. Mckenzie, J.C. Krepinsky, RhoA/Rho-kinase contribute to the pathogenesis of diabetic renal disease. Diabetes. 2008; 57:1683–92. 10.2337/db07-1149 18356410

[33] Kolavennu V, Zeng L, Peng H, Wang Y, Danesh FR. Targeting of RhoA/ROCK signaling ameliorates progression of diabetic nephropathy independent of glucose control. Diabetes. 2008; 57:714–23. 10.2337/db07-1241 18083785

[34] Rodriguez-Vita J, Ruiz-Ortega M, Rupérez M, Esteban V, Sanchez-López E, Plaza JJ, Egido J. Endothelin-1, via ETA receptor and independently of transforming growth factor-beta, increases the connective tissue growth factor in vascular smooth muscle cells. Circ Res. 2005; 97:125–34. 10.1161/01.RES.0000174614.74469.83 15976312

[35] Lee TM, Chung TH, Lin SZ, Chang NC. Endothelin receptor blockade ameliorates renal injury by inhibition of RhoA/Rho-kinase signalling in deoxycorticosterone acetate-salt hypertensive rats. J Hypertens. 2014; 32:795–805. 10.1097/HJH.0000000000000092 24463935

