# Supplementary material for: Endothelial cells secreted endothelin-1 augments diabetic nephropathy via inducing extracellular matrix accumulation of mesangial cells in ETBR-/- mice
Source: Aging (Albany NY). 2019 Mar 29;11(6):1804–20. doi: 10.18632/aging.101875 (PMC6461170; doi:10.18632/aging.101875)
Supplement: Supplementary Figure [file aging-11-101875-s001.pdf]

SUPPLEMENTARY MATERIAL

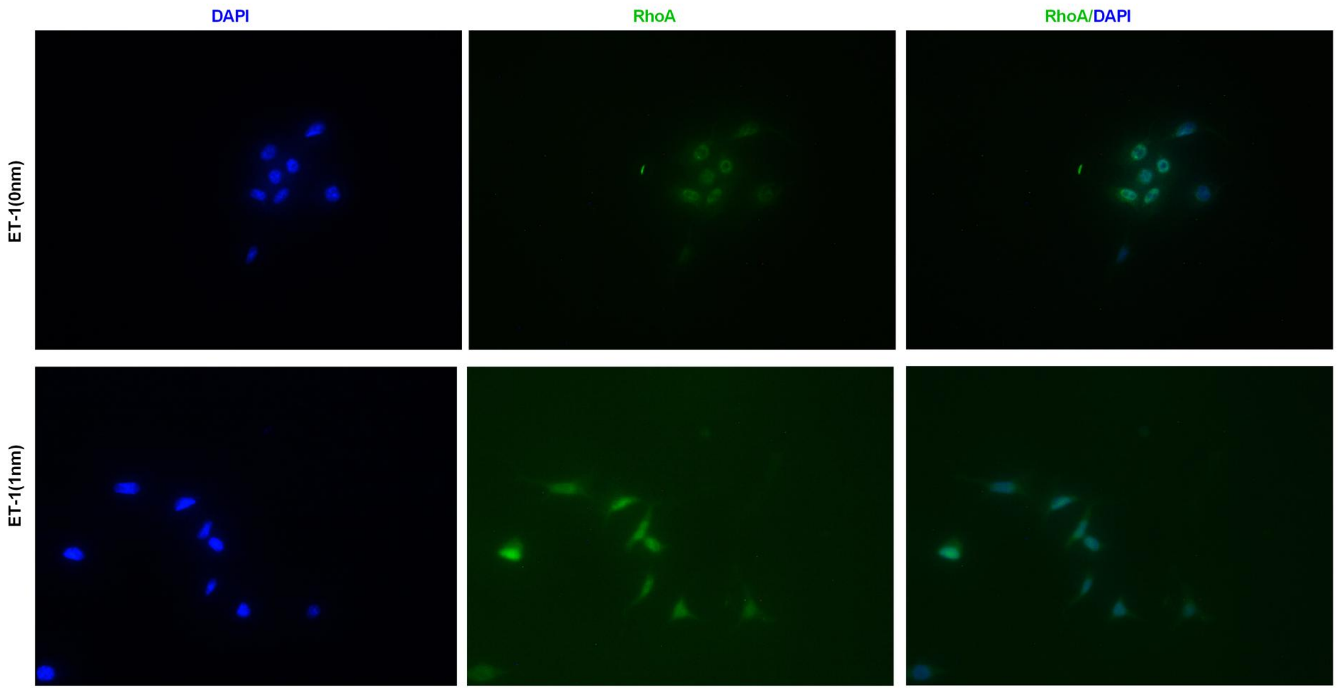

Supplementary Figure 1. RhoA diffused to cell membrane of mesangial cells. Mesangial cells were treated with ET-1 (0 nm) or ET-1 (1 nm), then immunofluorescence assay was used to determine whether RhoA diffused to cell membrane of mesangial cells after the treatment of ET-1.
